# A Complex Nutrient Exchange Between a Gall-Forming Aphid and Its Plant Host

**DOI:** 10.3389/fpls.2020.00811

**Published:** 2020-07-07

**Authors:** Xiaoming Chen, Zixiang Yang, Hang Chen, Qian Qi, Juan Liu, Chao Wang, Shuxia Shao, Qin Lu, Yang Li, Haixia Wu, Kirst King-Jones, Ming-Shun Chen

**Affiliations:** ^1^Research Institute of Resource Insects, Chinese Academy of Forestry, Kunming, China; ^2^Key Laboratory of Breeding and Utilization of Resource Insects of State Forestry Administration, Kunming, China; ^3^College of Horticulture and Landscape, Southwest Forestry University, Kunming, China; ^4^Department of Biological Sciences, University of Alberta, Edmonton, AB, Canada; ^5^Department of Entomology, Kansas State University, Manhattan, KS, United States

**Keywords:** galling aphid, closed horned gall, CO_2_ accumulation, honeydew, photosynthesis, glycometabolism, nutrient exchange

## Abstract

It has been a long-standing question as to whether the interaction between gall-forming insects and their host plants is merely parasitic or whether it may also benefit the host. On its host *Rhus chinensis*, the aphid *Schlechtendalia chinensis* induces the formation of closed galls, referred to as horned galls. Typically, mature aphid populations comprise thousands of individuals, which is sufficient to cause the accumulation of high CO_2_ levels in galls (on average 8-fold higher and up to 16 times than atmospheric levels). Large aphid populations also excrete significant amounts of honeydew, a waste product high in sugars. Based on ^13^C isotope tracing and genomic analyses, we showed that aphid-derived carbon found in CO_2_ and honeydew was recycled in gall tissues via photosynthesis and glycometabolism. These results indicated that the aphid-gall system evolved in a manner that allowed nutrient recycling, where the gall provides nutrients to the growing aphid population, and in turn, aphid-derived carbon metabolites provide a resource for the growth of the gall. The metabolic efficiency of this self-circulating system indicates that the input needed from the host plant to maintain aphid population growth less than previously thought and possibly minimal. Aside from the recycling of nutrients, we also found that gall metabolites were transported to other parts of the host plant and is particularly beneficial for leaves growing adjacent to the gall. Taken together, galls in the *S. chinensis*–*Rhus chinensis* system are highly specialized structures that serve as a metabolic and nutrient exchange hub that benefits both the aphid and its host plant. As such, host plants provide both shelter and nutrients to protect and sustain aphid populations, and in return, aphid-derived metabolites are channeled back to the host plant and thus provide a certain degree of “metabolic compensation” for their caloric and structural needs.

## Introduction

It has been long thought that gall-inducing insects reprogram the physiology of the host plant in a way that primarily benefits the growing insect colony (Larson and Whitham, 1991; Danks, 2002; Inbar et al., 2004; Koyama et al., 2004). The potential benefits of galls to galling insects include providing shelters and nutrients for insect growth and development, protecting the insect colony from predators, and reducing environmental physical threats such as sunlight, desiccation, or heavy rain (Price et al., 1987; Fernandes and Price, 1992; Brown et al., 1995; Quintero et al., 2014). Gall formation may have evolved as a mechanism to sequester galling insects, thus protecting other parts of the plant from potential damage (Price et al., 1987; Wool, 2004). Very little is known about potential benefits that gall insects may provide to the host plant. It is generally thought that the impact of gall insects on plant growth is negative (Price et al., 1987). However, some studies have shown that the relationship between gall inducers and host plants is not merely a simple parasitic/defensive relationship. Jasmonic acid (JA) plays an important role in regulating plant defense responses to phloem-feeding insects (Thompson and Goggin, 2006; Howe and Jander, 2008; Morkunas et al., 2011). However, JA levels do not increase significantly in horned galls when compared to control leaf samples (Wang et al., 2016), suggesting that the relationship between the gall and its host plant is benign. The gall-inducing species *Eurosta solidaginis* and *Gnorimoschema gallaesolidaginis* do not cause significant increases in defensive volatile emissions from the host, *Solidago altissima* (Tooker et al., 2008). In some cases, galled branches have more leaves than ungalled branches and tend to have more biomass (Kurzfeld-Zexer et al., 2010). Studies have shown that the overall metabolism of the gall-bearing plants appears to remain unaffected, except for stress-related changes at and near the locations where galls have developed (Raman, 2011). These findings suggest a more complex interaction between a gall-inducing insect and its host plant, as opposed to a simple parasitic/defensive relationship.

**FIGURE 1:**
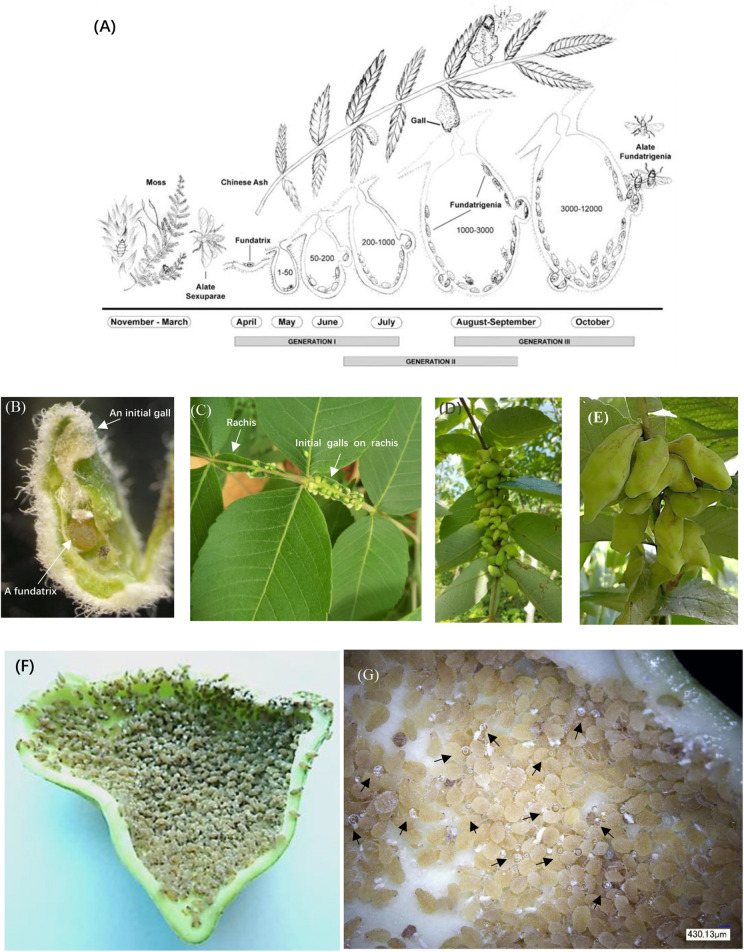
Life cycle and anatomical features of horned galls. **(A)** Life cycle of *Schlechtendalia chinensis*. *S. chinensis* is a heteroecious species and requires two host plant species, a moss species and the Chinese ash. After overwintering in moss, winged (alate) sexuparae fly to trunks of the primary host Chinese ash (*Rhus chinensis*), where they deposit male and female sexuales on the branch surface. This is the only sexuparous stage in its life cycle. After mating, each female produces a single apterous fundatrix, which initiates gall formation on the host tree. Within a gall, three generations of fundatrigenia are produced parthenogenetically from the fundatrix. Late in the season, winged fundatrigenia fly away from dehisced galls to the winter host (moss), where the life cycle is completed by the formation of parthenogenetically produced winged Sexuparae. **(B)** A fundatrix and its newly induced gall at the initial stage. **(C–E)** Gall development in different stages with **(C)** in July, **(D)** in August, and **(E)** in September. **(F)** High density of aphids living inside a late-stage gall. **(G)** Aphid-excreted honeydews inside a gall. Black arrowheads indicate honeydew droplets.

Here we used the *Schlechtendalia chinensis*–*Rhus chinensis* system to investigate potential benefits that may exist for either of the interacting organisms. The aphid *S. chinensis* induces the formation of Chinese horned gallnuts on the Chinese Ash shrub *R. chinensis*. *S. chinensis* has a complex life cycle with cyclical parthenogenesis where different generations reside on alternate hosts between shrub and moss (Figure 1A). The horned galls are enclosures that grow continuously from May to October and accommodate a growing colony of aphids until they reach a population size of several thousand individuals toward the end of the season (Figures 1B–F). The large size of outgrowth gall tissues and an insect population size up to 12,000 aphids (Shao et al., 2013) would suggest a heavy burden that the insect puts on its host plant. Interestingly, the volume of galls continually grows as the aphid population increases in size, while the host plant appears to have no drastic defensive response to aphids. Instead, the host plant continues to provide minerals and other nutrients for aphids and to protect them from natural enemy rather than to curb aphid population growth by shrinking galls or fallen leaves. This raises an interesting question as to why the host plant tolerates these substantial energy and material costs to support gall growth. Is there any potential benefit from galling insects to the host plant as well? If so, what could a large colony of aphids contribute to the host plant as compensation?

During the process of gall formation and development, aphids produce a range of metabolites as a result of feeding and excretion. A large aphid population in a closed gall will produce a high CO_2_ concentration and excrete a substantial amount of honeydew (Figure 1G). However, it is unclear whether any of these metabolic aphid by-products will benefit the host plant. Elevated levels of atmospheric CO_2_ directly affect plant physiology and have been shown to increase in photosynthetic activity and carbohydrate yield (Cure and Acock, 1986; Masle, 2000; Smith and Al, 2000; Dijkstra et al., 2002; Jablonski et al., 2002; Veteli et al., 2002; Will and Ceulemans, 2006). We wondered whether the high concentrations of CO_2_ would fuel metabolic processes of the host plant. Moreover, aphids excrete substantial amounts of honeydew, which was shown by Kutsukake et al. (2012) to be absorbed by the inner gall surface. This raises the question as to whether honeydew metabolites will be utilized by the host plant as an energy source to promote growth.

To understand the response host plants mount to counter an aphid attack and to examine whether there is nutrient exchange between aphids and their host plants, we measured a range of aphid by-products, including CO_2_ and honeydew excreted by aphids in galls, and analyzed the rates of photosynthesis and glycometabolism in gall tissues and leaves. We also monitored aphid-derived ^13^C-labeled metabolites and analyzed genes involved in photosynthesis and glycometabolism. In this report, we show that CO_2_ and honeydew from aphids are in fact beneficial for the host plant, and we present a complex nutrient exchange model describing the metabolic recycling between the gall insect and its host plant.

## Materials and Methods

### Gall Cultivation

All experiments were carried out on fresh galls induced by the aphid *Schlechtendalia chinensis* on *R. chinensis* cultivated in the testing site (N 28° 06’ E 104° 22’ H 820 m) located at the gall cultivation area in Yanjin County, Yunnan Province, Southwest China.

### Measurement of CO_2_ Concentrations in Aphid Galls

We used a portable photosynthesis system (Li-6400 Li-COR, United States) with a conifer chamber attachment to measure CO_2_ levels inside galls. Measurements were conducted with fresh galls directly in the field. CO_2_ concentrations were determined individually. Specifically, a fresh gall collected from a branch of a host tree was placed into the conifer chamber. The chamber was closed to avoid any gas leakage. A dissecting needle was pre-inserted into the chamber through the plastic cushion with its sharp point near the gall wall. To measure CO_2_ inside the gall, the needle was carefully pressed to penetrate the gall wall to release gases from the inside of the gall. When CO_2_ reading became steady in the chamber, the measurement of CO_2_ was recorded. Thirty-two individual galls were measured. At the same time, CO_2_ in the atmosphere was recorded at the same location (*n* > 35). The CO_2_ concentration in a gall was calculated using the following formula:

CO2 concentration in gall =(CO2 reading in a gall − CO2reading in the air) × Conifer Chamber volume (266.86 cm3)/gallvolume.

### Gall Volume and Aphid Density Determination

A small hole was introduced on the top of a gall. Individual aphids were removed through the hole and counted. The emptied gall was immersed into water, and the displaced water volume was measured with a graduate cylinder. Average gall volume was derived from 32 galls. Aphid density was calculated by the following formula:

a⁢p⁢h⁢i⁢d⁢d⁢e⁢n⁢s⁢i⁢t⁢y=t⁢o⁢t⁢a⁢l⁢n⁢u⁢m⁢b⁢e⁢r⁢o⁢f⁢a⁢p⁢h⁢i⁢d⁢s⁢i⁢n⁢a⁢g⁢a⁢l⁢l/g⁢a⁢l⁢l⁢v⁢o⁢l⁢u⁢m⁢e.

### Photosynthetic Rate Measurements

Gall photosynthesis: Photosynthetic rates of galls and leaves were measured by a Li-6400 plant photosynthesis System OPEN 6.3.2 (The Gene Co., Ltd., United States). Samples were put in the external areatus chamber with the controllable light clusters under the light intensity controlled in two conditions 500 μmol m-2 s-1 and 0 μmol m-2 s-1, respectively.

The surface area of a gall was calculated based on sunlight-irradiable area. At the height of the control light source, a vertical photograph was taken for the gall. The software AutoCAD 2010 (Autodesk Computer Aided Design, United States) was used to measured and calculate the gall projective area and the surface area of the leaf chamber in the photograph. The gall surface area exposed to the light was calculated by the following formula:

$$\begin{array}{l} The\;gallnut\;surface\;area\;exposed\;to\;the\;light=surface\;area\;of\\ leaf\;chamber\times gallnut\;projective\;area\;in\;photograph/surface\\ area\;of\;leaf\;chamber\;in\;photograph. \end{array}$$

To determine the relationship between photosynthesis rates and gall sizes, photosynthesis rates were measured for a total of 30 individual galls in different sizes.

Leaf photosynthesis: The surface area of a leaf for photosynthesis was measured directly. To determine the photosynthesis rates in galls at different developmental stages, photosynthesis rates were measured from 25 galls and 16 leaves in July, August, and September.

### Selection and Measurement of Leaves Near a Gall and Leaves in Trees Free of Galls

The selection of trees with and without galls was carried out randomly in the nursery of our experimental station site in September. All trees in the nursery were at the same age and under similar growth conditions. Branches of gall-bearing trees and gall-free trees were chosen for leaf measurement. Each sample contained more than 60 branches (a cluster of pinnately compound leaves) and respectively 559 leaves with gall and 543 leaves without gall.

### Honeydew Excretion Analysis and Absorption

To analyze the composition of honeydew, honeydew droplets were collected directly from different galls with a pipette. Sugar compositions were analyzed using a High Performance Liquid Chromatography instrument (Agilent 1200, United States, Vólkl et al., 1999). A chromatographic column (5 μm, 250 × 4.6 mm, Agilent Co., Ltd., United States) was used for ZORBAX carbohydrate analysis. The mobile phase was 75 acetonitrile: 25 water (V/V). Solvents were filtered by 0.45 μm membrane before use. Flow rate was 1.0 ml/min under 35°C. A refractive index detector (RID) was used and equilibrated under 35°C as well. For each analysis, 15 μl honeydew was subjected to separation through the column. Individual sugar species, including fructose, mannose, glucose, sucrose, trehalose, xylose, galactose, rhamnol, and mannitol, were identified based on their retention times. Each assay was repeated three times independently.

To analyze absorption of sugars in honeydew by gall tissues, galls were collected together with their host branches and were carried back to the laboratory. The branches were put into containers with water. A hole was introduced on the top of each gall. After removing aphids from galls, 0.5 ml of 2 or 5% of a sugar solution containing fructose, glucose, and sucrose at a ratio of 4:3:1 (based on their ratio in honeydew) was injected into each gall, and the opening on the gall was sealed immediately with wax. At 4, 6, 8, or 10 h after the injection, galls were opened and the leftover sugar solution was observed. Complete absorption was recorded if no leftover sugar solution was found inside a gall. Absorption rates of gall tissues were calculated by the following formula: Complete absorptive rate (%) = complete absorptive samples/observed samples × 100%. Water was used as a control. Each measurement was repeated 10 times.

### Isotope Tracing Analyses

All isotope tracing experiments were done on trees in the field directly. For ^13^CO_2_ tracing in galls, ^13^CO_2_ gas (99% ^13^C) was injected into galls growing on a tree using a 5-ml injector (inner diameter 11.99 mm). A single injection of 2.0 ml ^13^CO_2_ gas was carried out at 9:00 am each day, and the injection was continued for 3 consecutive days. For ^13^CO_2_ tracing in leaves, ^13^CO_2_ gas was injected into a sealed bag (10 cm × 15 cm), which covered a leaf. A single injection of 5.0 ml ^13^CO_2_ gas was made at 9:00 am each day. The injection was continued for 5 consecutive days. For ^13^C-glucose tracing in galls, a micro-injector with the inner diameter 0.41 mm (G22) was used to inject 0.2 ml of ^13^C-glucose solution (5% ^13^C) into a gall at 9:00 am each day. Injection was continued for 3 consecutive days. All samples were collected and preserved in liquid nitrogen until analysis.

To monitor isotope distribution, tissue samples were collected at different times and were dried thoroughly at 70°C. The dried samples were then ground into powder. Thirty milligrams of the powder for each sample was subjected to isotope analysis. Samples were individually burned at high temperature to generate CO_2_ by using a Flash 2000 EA-HT Elemental Analyzer (Thermo Fisher Scientific, Inc., United States). The amounts of ^13^C and ^12^C were measured using an Isotope Ratio Mass Spectrometer (DELTA V Advantage, Thermo Scientific Inc., United States). The ratios of δ^13^C were calculated by comparing with the international standard (Pee Dee Belemnite = PDB). ^13^C absolute abundance (δ^13^C) was calculated using the following formula:

$$\begin{array}{l} \delta^{13}C(‰)={(}^{13}C{/}^{12}C\_sample{/}^{13}C{/}^{12}C\_PDB\;-\;1)\times 1000\\ \text{Measurement accuracy}:\;\delta^{\text{13}}\text{C}:\;\pm \;<\;0.\text{1}‰\\ \text{All measurements were repeated three times}. \end{array}$$

### Transcriptomic Sequencing and Analyses

Gall and leaf samples were randomly collected from host trees in the field. Then, samples were immediately frozen in liquid nitrogen and stored at −80°C. Total RNA was extracted respectively by TaKaRa MiniBEST Plant RNA Extraction Kit (TaKaRa, China). The concentration of total RNA was determined by NanoDrop 2000 (Thermo Fisher Scientific, United States). A total amount of 3 μg RNA per sample was used for transcriptomic analysis. mRNA was purified from total RNA using magnetic beads coated with oligo-dT (Sangon Biotech, China). Fragmentation was carried out using divalent cations under elevated temperature in NEBNext First Strand Synthesis Reaction Buffer (5X). First-strand cDNA was synthesized using a random hexamer primer and M-MuLV Reverse Transcriptase (RNase H). Second-strand cDNA synthesis was subsequently performed using DNA Polymerase I and RNase H. Remaining overhangs were converted into blunt ends via exonuclease/polymerase activities. After adenylation at 3′-ends of DNA fragments, NEBNext Adaptor with hairpin loop structure was ligated to prepare for hybridization. In order to select cDNA fragments of preferentially 150–200 bp in length, the library fragments were purified with AMPure XP system (Beckman Coulter, Beverly, United States). Then, 3 μl USER Enzyme (NEB, United States) was used with size-selected, adaptor-ligated cDNA at 37°C for 15 min followed by 5 min at 95°C before PCR. PCR was performed with Phusion High-Fidelity DNA Polymerase, Universal PCR primers, and Index (X) Primer. At last, PCR products were purified (AMPure XP system) and library quality was assessed on the Illumina HiSeq 2000 platform (BGI, Shenzhen, China). Nine transcriptomic libraries (three different types of tissues with three repeats for each) were made in this study. Libraries were generated using a NEBNext^®^ Ultra^TM^ RNA Library Prep Kit from Illumina^®^ (NEB, United States) following the manufacturer’s protocol. Different barcodes were added to different samples for later sorting.

After sequencing, clean reads were obtained by removing reads containing adapter, reads with more than 5% N base, and reads of low quality. At the same time, Q20, Q30, GC content, and sequence duplication level of the clean data were calculated. All the downstream analyses were based on clean data with high quality. Transcriptome assembly was accomplished using Trinity (Grabherr et al., 2011) with min_kmer_cov set to 2 by default and all other parameters set default. Each transcriptomic analysis was repeated three times independently.

### Salicylic Acid (SA) Measurement During Gall Growth and Aphid Population Expansion

During gall development from April to October, SA in gall and leaf tissues were measured along with gall size and aphid population size within a gall every 10–20 days. Samples were collected from host plants in the field and then immediately frozen in liquid nitrogen and stored at −80°C. SA was extracted according to the methods of Raskin et al. (1989) and Wang et al. (2002). After centrifugation, the supernatant was passed through a Sep-Pak C_18_ cartridge (Waters). For each sample, 300 μl exude solution was dried under N_2_ steam at 40°C and then was dissolved in 300 μl PBS for determination of SA using direct competitive ELISA.

The extraction and purification of SA was carried out according to the instruction of ELISA kits. SA in galls and leaves were measured using ELISA kits (purchased from China Agricultural University, Beijing, China, Wang et al., 2016). All standards and samples were added in duplicate to the Microelisa stripplate. Standard wells and testing sample wells were set, and the 50-μl standard was added to the standard well. A 10-μl sample was added, and 40 μl diluent was added to the testing sample well. Nothing was added to the blank. 100 μl of HRP-conjugate reagent was added to each well, covered with an adhesive strip and incubated for 60 min at 37°C. Each well was aspirated and washed, repeating the process four times for a total of five washes. A squirt bottle was used to wash by filling each well with a wash solution (400 μl). After the last wash, any remaining wash solution was removed by aspirating or decanting. The plate was inverted and blotted against clean paper towels. 50 μl chromogen solution and 50 μl of chromogen solution B were added to each well, which were gently mixed and incubated for 15 min at 37°C without light. A 50-μl stop solution was added to each well, and the color in the wells changed from blue to yellow. The optical density (O.D.) at 450 nm was checked using a microtiter plate reader within 15 min. Each assay was replicated independently for three times.

### Statistical Analysis

Statistical analysis was conducted using the IBM SPSS Statistics 19 software. The least significant difference (LSD) test was carried out for multiple comparisons among groups, and the independent-samples *T*-test was conducted for comparisons between two groups.

## Results

### CO_2_ Concentrations Are Highly Elevated in the Gall Interior

**FIGURE 2:**
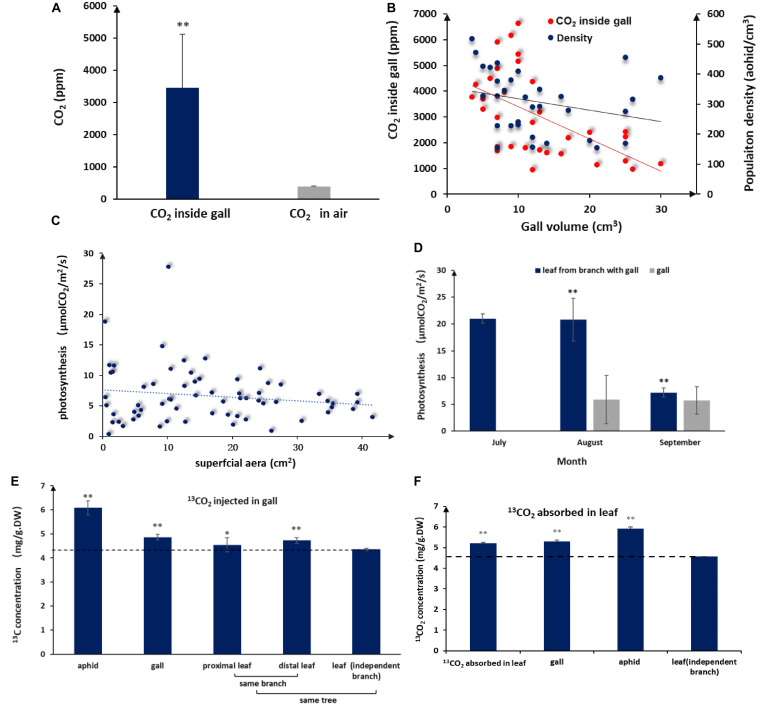
Elevated CO_2_ and photosynthesis levels in galls and leaves. **(A)** Average interior and exterior CO_2_ concentrations (*n* = 32, error bars denote SD). **(B)** Population density of aphid and CO_2_ in the volume of different galls. **(C)** Photosynthesis rates of gall tissues in different sizes (*n* = 66). **(D)** Photosynthesis rates in galls and leaves during gall development (no photosynthesis data available for July because galls are too small). **(E)**^13^C distribution in aphids and different plant tissues after 5 days of consecutive ^13^CO_2_ injections into the gall (DW = dry weight, LSD-test). **(F)** ^13^C distribution in aphids and different plant tissues after 5 days of consecutive absorption of ^13^CO_2_ in a leaf close to a gall (LSD test). ** represent *P* < 0.01, * represent *P* < 0.02.

Aphid populations in closed galls comprise thousands of individuals, and consequently, aphid by-products are bound to accumulate inside galls. When we measured CO_2_ concentrations inside and outside of galls, we found that CO_2_ concentrations in the gall interior were on average 8-fold higher (3459.69 ± 1655.65 ppm) and could reach levels as high as 16 times (6640.35) compared to atmospheric levels (398.88 ± 8.08 ppm) (Figure 2A). We observed a negative linear relationship between gall volume and CO_2_ concentrations, with CO2 concentrations declining as gall size increased (Figure 2B).

### Aphid-Derived CO_2_ Molecules Are Reused

Because the surface of a gall is green, it was possible that the gall itself is photosynthetically active, in which case we would expect that interior CO_2_ could directly fuel photosynthesis in gall tissues. When we measured photosynthesis rates in the gall, we found that gall tissues had measurable levels of photosynthesis even though photosynthesis per unit of gall tissues was lower than that of leaves (Figures 2C,D). In addition, photosynthetic activity in gall tissues declined slightly as gall size increased (Figure 2C), consistent with CO_2_ concentration change in galls, suggesting that photosynthesis in gall tissues is closely related to CO_2_ concentrations inside a gall.

To further test whether aphid-derived CO_2_ was reused via photosynthesis, we injected ^13^CO_2_ directly into galls and, then, tracked ^13^C accumulation in the gall tissues, aphids, and neighboring leaf tissues 3 days after the last ^13^CO_2_ injection. Accumulation of ^13^C was detected in gall tissues, gall-residing aphids, and leaves surrounding the gall. The highest ^13^C accumulation was found in aphids, but significant amounts of ^13^C were also detected in gall tissues, proximal leaf tissues, and distal leaf tissues (Figure 2E). These results demonstrated that CO_2_ accumulated in galls could contribute to photosynthesis in gall tissues and that gall-produced metabolites can be used by aphids and transported to adjacent tissues. On the other hand, after ^13^CO_2_ was absorbed by leaves for 3 days, we detected ^13^C in gall tissues and aphids (Figure 2F), indicating that photosynthates in leaves were transported to gall tissues and used by aphids. These results have shown that photosynthates between gall and leaf areinter-transportable.

### Sugars in Honeydew Can Be Recycled in Galls and Nearby Leaf Tissues

**FIGURE 3:**
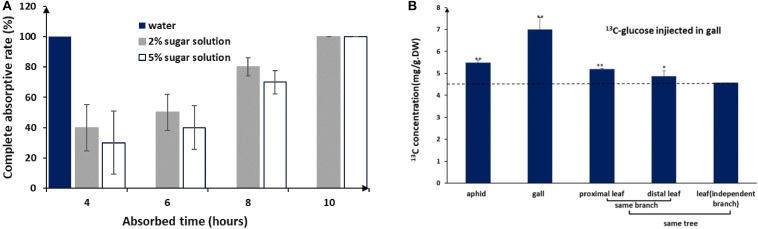
Honeydew absorption and ^13^C-glucose tracing. **(A)** Completed absorption rate of water and sugar solutions by gall tissues. **(B)** ^13^C distribution in aphids and different plant tissues after 3 days of consecutive injections of ^13^C-glucose into the gall (LSD test). ** represent *P* < 0.01, * represent *P* < 0.02.

Like other aphids, *S. chinensis* secreted substantial amounts of honeydew, which was composed of fructose (41.1 ± 8.8%), glucose (34.9 ± 7.5%), sucrose (9.1 ± 0.1%), and five unknown sugars (14.9%). Sugar concentrations in honeydew were 2–3%. In our mimic absorption test, 2–5% sugar solutions could be completely absorbed by the inner wall of galls within 10 h (Figure 3A). When ^13^C-labeled glucose was injected into galls, ^13^C-glucose was detected in gall tissues, gall-residing aphids, and nearby leaf tissues (Figure 3B), suggesting that aphid-derived sugars can be absorbed by gall tissues, reused by aphids, and transported to other parts of the host plant.

### Transcriptomic Profiling of Key Genes Involved in Photosynthesis and Sugar Metabolism

**FIGURE 4:**
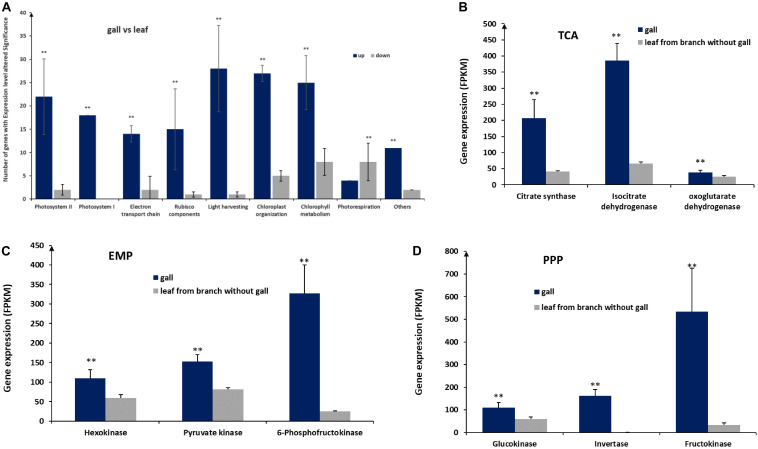
Expression of genes involved in photosynthesis and glycometabolism. **(A)** Expression of genes in nine pathways associated with photosynthesis. **(B)** Expression of genes of the tricarboxylic acid (TCA) cycle, encoding citrate synthase, isocitrate dehydrogenase, and oxoglutarate dehydrogenase. **(C)** Expression of genes encoding hexokinase, pyruvate kinase, and 6-phosphofructokinase acting in the Embden–Meyerhof–Parnas pathway (EMP). **(D)** Expression of genes acting in the pentose photosphate pathway (PPP), encoding glucokinase, invertase, and fructokinase. ** represent significant difference, *P* < 0.01.

To identify important gene expression differences between gall and leaf tissues, we reanalyzed transcriptomic data comparing gall and leaf samples. From this analysis, we identified 166 transcripts associated with photosynthesis in gall tissues^1,2^. The genes corresponding to these transcripts fell into nine categories, including photosystem I, photosystem II, electron transport chain, Rubisco components, light harvesting, chloroplast organization, photorespiration (Figure 4A). Except for genes linked to photorespiration, which reduce photosynthesis efficiency, genes from the other eight categories are required for photosynthesis. Interestingly, on average, genes from all categories were expressed at significantly higher levels in gall tissues than in control leaf tissues, except for genes in the photorespiration category. The upregulation of photosynthesis genes and the downregulation of photorespiration genes suggested increased photosynthesis rates in gall tissues relative to leaf tissues.

We also found that 199 genes related to sugar metabolism were significantly upregulated in galls, including genes encoding key enzymes, such as citrate synthase, isocitrate dehydrogenase, and oxoglutarate dehydrogenase in tricarboxylic acid cycle (TCA), hexokinase, pyruvate kinase, and 6-phosphofructokinase in the Embden–Meyerhof–Parnas pathway (EMP), and encoding glucokinase, invertase, and fructokinase in the pentose photosphate pathway (PPP) (Figures 4B–D), suggesting that the higher sugar metabolism that occurred in galls could be used for more energy and necessary intermediates for the growth and development of the galls.

### Characterization of Physiological and Growth Parameters of Galls and Leaves During Gall Development

**FIGURE 5:**
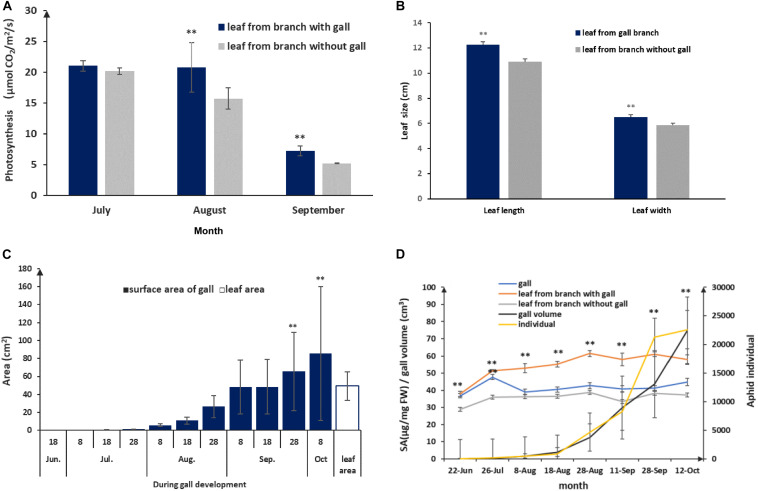
Physiological and growing characterization of gall and leaf during gall development. **(A)** Photosynthesis rate comparison between leaves with a nearby gall and leaves without adjacent gall. **(B)** Leaf sizes of leaves without adjacent and leaves with a nearby gall in September. **(C)** Leaf area and area of the gall surface during gall development. **(D)** SA response in gall and leaf during aphid population rising and gall growth. ** represent *P* < 0.01.

Host plants appeared to grow unperturbed in the presence of gall growth. To understand how the host plant responds to gall growth, we measured photosynthetic rates in leaves collected from branches with and without galls. We found that there were no differences in the early stages of gall formation. However, there were significant differences in the middle (August) and late stages (September), where the photosynthetic rates of leaves surrounding galls were higher compared to leaves with no nearby galls. In addition, we found that the average leaf size exceeded that of leaves from gall-fee branches (*p* < 0.01; Figures 5A,B). From July to September, galls gradually increased in size and the area of the gall surface area grew faster than the leaf surface area in late August (*p* < 0.01) and exceeded 80 cm^2^ in mature galls (Figure 5C). This suggested that the larger gall area would promote a higher photosynthetic rate to support gall growth. During gall development, gall growth was relatively slow prior to August, but salicylic acid (SA) concentrations were significantly increased compared to leaf samples from gall-free branches (*p* < 0.01, Figure 5D). This suggested that the observed increase in SA represents the host plant defense response to the growing aphid population. After the middle of August, despite aphid populations reaching maximum levels, the SA levels in leaves adjacent to galls showed only a mild increase, whereas SA in the gall declined and showed no difference compared to leaves not close to a gall (Figure 5D). Taken together, this showed that a balance could be established between galls and aphid as the galls and aphid populations matured.

## Discussion

We described here the first and the only known example of an animal species that lives above ground at an elevated level of CO_2_ above 3000 ppm in its natural habitat. Tolerance to a high CO_2_ environment may be of adaptive significance to *S. chinensis*, as high CO_2_ levels have been shown to boost population size in aphid species that have low nitrogen requirements (Bezemer and Jones, 1998; Holopainen, 2002; Newman et al., 2003; Chen et al., 2004; Peltonen et al., 2006). The fact that a large population of aphids can thrive in closed galls under high CO_2_ suggests that the aphid species has adapted to an environment with elevated levels of CO_2_.

Elevated levels of atmospheric CO_2_ directly affect plant physiology and have been shown to increase photosynthetic activity and carbohydrate yields of some trees (Dijkstra et al., 2002; Will and Ceulemans, 2006; Tissue et al., 2010; Grombone-Guaratini et al., 2013). Our ^13^CO_2_ isotopic tracing detected ^13^C in gall, and transcriptomic data showed enhanced expression of 166 genes involved in photosynthesis. These results suggested that high CO_2_ may have actually fueled photosynthesis in gall tissues. High level of CO_2_ in galls could stimulate photosynthesis in gall tissues, even though the rates of photosynthesis per unit of gall tissues were lower than those of leaves. Lower photosynthesis rates in gall tissues in comparison with leaves have been observed in other systems and are likely due to alteration in chlorophyll metabolism (Huang et al., 2014). However, the horned gall system is unique because of the huge surface area of a gall. In addition, a large fraction of the chlorophyll in the light harvesting antennae is superfluous for driving the light reactions (Evans, 1989; Anderson et al., 1995; Martin et al., 2007). A combination of high CO_2_ concentrations and enlarged surficial areas of galls could compensate the lack of photosynthetic pigments. Some studies suggested that photosynthetic rates were reduced in leaves close to a gall (Florentine et al., 2005; Patankar et al., 2011; Kar et al., 2013; Kmieć et al., 2018) while other reports showed increased photosynthesis in gall-associated leaves (Fay et al., 1993; Dorchin et al., 2006). The difference could be explained by the diverse taxa that were used in these studies and represent different microstructures of different gall types. In the horned gall system, an aphid population measuring thousands of individuals will produce high CO_2_ concentrations and as such promote photosynthesis in leaves of the host plant.

In a closed gall, except for high levels of CO_2_, aphids secrete large amounts of honeydew. In open galls, aphids can push honeydew droplets out from the galls (Aoki and Kurosu, 1988; Benton and Foster, 1992; Pike et al., 2002). However, honeydew cannot be removed from closed galls. Our observation here along with a previous report (Kutsukake et al., 2012) indicated that honeydew droplets were absorbed by the inner wall of galls. Sugars present in honeydew are likely reused by gall tissues via sugar metabolism. Consistent with this possibility, our transcriptomic data showed that the genes encoding key enzymes in TCA, EMP, and PPP were significantly upregulated in gall tissues compared to control leaf tissues. Furthermore, our isotope tracing of labeled glucose showed that ^13^C was detected in gall tissues, suggesting that simple sugars in honeydew were reused in galls.

In the *S. chinensis*–*Rhus chinensis* system, both CO_2_ and honeydew from aphids can be recycled effectively within galls and provided nutrition for aphids. As a result, a nutrient recycling system evolved between gall tissues and aphids. The high metabolic efficiency of galls means that minimum input is needed from the host plant to maintain an aphid population within a gall. In a traditional source (surrounding leaves)–sink (galls) model (Larson and Whitham, 1997), the leaves nearby galls would do poorly since significant amounts of photoassimilates and other nutrients would be transported to the galls for gall development and aphid consumption. Remarkably, we observed the opposite phenomenon with *S. chinensis*-induced galls: the leaves next to galls were not negatively affected at all. In fact, the nearby leaves did better than leaves on trees without any galls, with higher photosynthesis rates and bigger leaf sizes (Figures 5A,B), Dorchin et al. (2006) observed more branches on trees with galls compared with those on trees without any galls. Kurzfeld-Zexer et al. (2010) reported that galled branches have in fact more leaves and tend to gain more biomass than ungalled branches.

Except for recycling of nutrients and metabolites, part of the gall tissue nutrients were likely transported to adjacent leaves, which is based on the ^13^C isotope-tracing tests we conducted. Specifically, these showed that ^13^CO_2_ and ^13^C-glucose in galls were transported to leaves adjacent to galls. The host plant is connected to the gall via the inter-connective transport system (Liu et al., 2014), which states that the relationship between source and sink is interchangeable at different stages. At early developmental stages, individual galls may act mainly as a nutrient sink initially because of aphid uptake. Photosynthetic metabolites produced in leaves are transported to and accumulate in galls, thus providing nutrition and structural support for aphid development and gall growth. As growth continues, galls increase in size, and at the end of the fast growth stage (the end of August to the beginning of October), the average total surface area of a gall exceeds that of a leaf (Figure 5C). At this time, galls may serve as the main source to provide nutrients to aphids. Our isotopic tracing experiments indicated that part of the nutrients in a gall can inversely flow to circumjacent leaves. This flotation could be driven by the evaporation of the leaves because leaf rachis wings where galls are attached provide important channels for water to be conveyed to leaves (Lu et al., 2019). The inverse nutrient flow from a gall to the surrounding leaf may be responsible for the observed higher rates of photosynthesis and bigger sizes of leaves nearby galls.

In the *S. chinensis*–*R. chinensis* system, galls represent highly specialized structures that serve as a nutrient exchange hubs. At the beginning of gall development, namely from April to July, nutrients required for aphid population expansion and gall growth are primarily produced in the surrounding leaves. During this stage, salicylic acid (SA) increased significantly in galls and nearby leaves (Figure 5D), but responses to jasmonic acid (JA) in galls and nearby leaves were lower than in leaves not associated with galls (Wang et al., 2016). To defend against an aphid attack, high concentrations of tannin accumulate in galls (Chen et al., 2018). Bernays and Woodhead (1982) and Bernays et al. (1983) reported that tannin may serve as a nutrient for phytophagous insects. In our transcriptomic *S. chinensis* data set, we found three laccases, raising the possibility that these enzymes metabolize tannin so that it can be utilized as a nutrient for aphids. After July, galls grow rapidly and aphids in a gall reproduce rapidly as well up to thousands (Figure 5D; Shao et al., 2013). This large population produced high levels of CO_2_ and substantial amounts of honeydew. An enlarged gall surface takes in more sunlight and high level of CO_2_ appears to stimulate photosynthesis in gall tissues. In addition, sugars found in honeydew appear to provide energy to promote gall growth. This intertwined interaction between aphids and galls promotes the growth of both, resulting in gall development and population increase. In the recycling of nutrients, the aphid metabolites are reused in galls and may compensate, at least in part, for the consumption metabolites that were used for gall growth and aphid development. This recycling effectively reduces the requirement of photosynthates from surrounding leaves.

We found that SA abundance in galls declined drastically after July and maintained low levels until galls matured (Figure 5D), implying that a balance was reached between aphid’s attack and the defense response from the host. Similarly, SA responses in leaves tended to be mild (Figure 5D) but were slightly higher likely due to the biotic stress caused by aphid feeding. Except for nutrient recycling, photosynthates in galls were also transferred to surrounding leaves, contributing to leaf growth. Although the benefit of photosynthates from galls was difficult to quantify, we have little doubt that these nutrients are beneficial to host plant growth. In fact, comparing leaves from gall-free trees to leaves close to a gall not only appeared to show no sign of negative impact but even did better overall (Figures 1D,E; Kurzfeld-Zexer et al., 2010; Raman, 2011).

**FIGURE 6:**
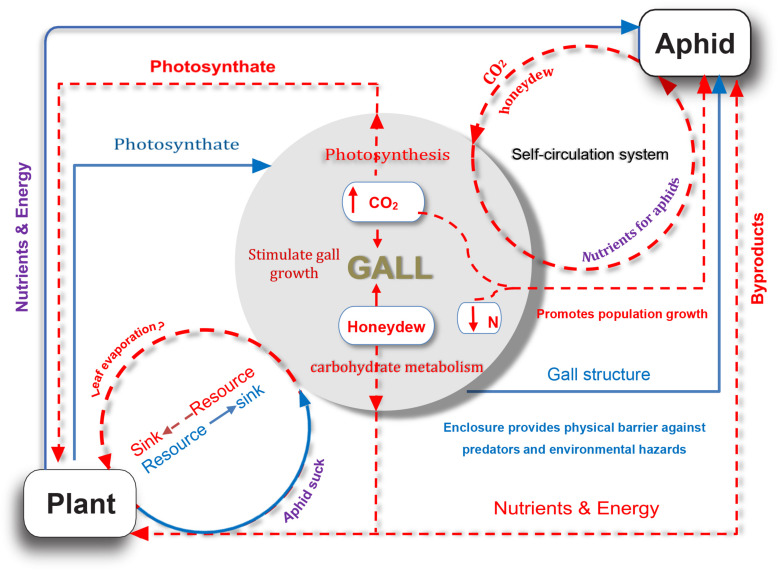
A model for nutrient exchange between host plant and gall-forming aphid colony. Solid blue lines represent that the host plant is beneficial for aphid; dotted orange red lines indicate potential indirect benefits from aphid to host plant.

Our study provided several lines of evidence for the complex nutrient exchange between a galling insect and its host plant, namely, the *S. chinensis*–*R. chinensis* system. In this system, the host plant providing shelters (galls) and nutrients for aphids (Larson and Whitham, 1991, 1997; Wool, 2004), and in return metabolites from aphids, compensated the host plant via photosynthesis and sugar metabolism (Figure 6). As such, this feedback mechanism alleviated the metabolic damage to the host by the aphids and reduced the impact of parasitic aphids on the plant to a degree where there was no visible harm to the host plant. This suggests a balanced system that developed well in the interaction of a parasite–host.

## Data Availability Statement

The datasets generated for this study can be found in the https://www.ncbi.nlm.nih.gov/sra/?term=PRJNA483267; https://www.ncbi.nlm.nih.gov/bioproject/PRJNA482730.

## Author Contributions

XC designed the experiments, performed the experiment of isotope tracing and biomass, and wrote the manuscript. ZY, CW, and YL detected CO_2_. CW, JL, and QL performed the experiments of photosynthesis, isotope tracing, and biomass. HC, QQ, and M-SC performed the transcriptomic sequencing and analyses. SS and HW investigated gall development and honeydew analysis and absorption. M-SC and KK-J analyzed the data and revised the manuscript. All authors read and approved the final manuscript.

## Conflict of Interest

The authors declare that the research was conducted in the absence of any commercial or financial relationships that could be construed as a potential conflict of interest.

## Footnotes

1 https://www.ncbi.nlm.nih.gov/sra/?term~=~PRJNA483267

2 https://www.ncbi.nlm.nih.gov/bioproject/PRJNA482730

## References

[B1] Anderson J. M. Chow W. S. Park Y. I. (1995). The grand design of photosynthesis: Acclimation of the photosynthetic apparatus to environmental cues^∗^. *Photosynth. Res.* 46 129–139. 10.1007/bf00020423 24301575

[B2] Aoki S. Kurosu U. (1988). Pemphigus “soldiers” and a defence of the generation-packing hypothesis: a response to itô and akimoto. *J. Ethol.* 6 65–67. 10.1007/BF02348865

[B3] Benton T. G. Foster W. A. (1992). Altruistic housekeeping in a social aphid. *Proc. R. Soc. B* 247 199–202. 10.1098/rspb.1992.0029

[B4] Bernays E. A. Chamberlain D. J. Woodhead S. (1983). Phenols as nutrients for a phytophagous insect *Anacridium melanorhodon*. *J. Insect Physiol.* 29 535–539. 10.1016/0022-1910(83)90085-9

[B5] Bernays E. A. Woodhead S. (1982). Plant phenols utilized as nutrients by a phytophagous insect. *Science* 216 201–203. 10.1126/science.216.4542.201 17736254

[B6] Bezemer T. M. Jones T. H. (1998). Plant-insect herbivore interactions in elevated atmospheric CO_2_: quantitative analyses and guild effects. *Oikos* 82, 212–222. 10.2307/3546961

[B7] Brown J. M. Abrahamson W. G. Packer R. A. Way P. A. (1995). The role of enemy escape in a gallmaker host-plant shift. *Oecologia* 104 52–609. 10.2307/4221078 28306913

[B8] Chen F. J. Wu G. Ge F. (2004). Impacts of elevated CO_2_ on the population abundance and reproductive activity of aphid Sitobion avenae Fabricius feeding on spring wheat. *J. Apply Entomol.* 128 723–730. 10.1111/j.1439-0418.2004.00921.x

[B9] Chen H. Liu J. Cui K. Lu Q. Wang C. Chen X. M. (2018). Molecular mechanisms of tannin accumulation in rhus galls and genes involved in plant-insect interactions. *Sci. Rep.* 8:9841. 10.1038/s41598-018-28153-y 29959354 PMC6026138

[B10] Cure J. D. Acock B. (1986). Crop responses to carbon dioxide doubling: a literature survey. *Agricult. For. Meteorol.* 38 127–145. 10.1016/0168-1923(86)90054-7

[B11] Danks H. V. (2002). Modification of adverse conditions by insects. *Oikos* 99 10–24. 10.2307/3547747

[B12] Dijkstra P. Hymus G. Colavito D. Vieglais D. A. Cundari C. M. Johnson D. P. (2002). Elevated atmospheric CO_2_ stimulates aboveground biomass in a fire-regenerated scrub-oak ecosystem. *Glob. Change Biol.* 8 90–103. 10.1046/j.1354-1013.2001.00458.x

[B13] Dorchin N. Cramer M. D. Hoffmann J. H. (2006). Photosynthesis and sink activity of wasp-induced galls in *Acacia pycnantha*. *Ecology* 87 1781–1791. 10.1890/0012-9658(2006)87[1781:pasaow]2.0.co;2 16922327

[B14] Evans J. R. (1989). Partitioning of nitrogen between and within leaves grown under different irradiances. *Aust. J. Plant Physiol.* 16 533–548. 10.1071/pp9890533

[B15] Fay P. A. Hartnett D. C. Knapp A. K. (1993). Increased photosynthesis and water potentials insilphium integrifoliumgalled by cynipid wasps. *Oecologia* 93 114–120. 10.1007/bf00321200 28313783

[B16] Fernandes G. W. Price P. W. (1992). The adaptive significance of insect gall distribution: survivorship of species in xeric and mesic habitats. *Oecologia* 90 14–20. 10.1007/bf00317803 28312265

[B17] Florentine S. K. Raman A. Dhileepan K. (2005). Effects of gall induction byepiblema strenuanaon gas exchange, nutrients, and energetics inparthenium hysterophorus. *BioControl* 50 787–801. 10.1007/s10526-004-5525-3

[B18] Grabherr M. G. Haas B. J. Yassour M. Levin J. Z. Thompson D. A. Amit I. (2011). Full-length transcriptome assembly from rna-seq data without a reference genome. *Nat. Biotechnol.* 29 644–652. 10.1038/nbt.1883 21572440 PMC3571712

[B19] Grombone-Guaratini M. T. Gaspar M. Oliveira V. F. Torres M. Aidar M. P. M. (2013). Atmospheric CO_2_ enrichment markedly increases photosynthesis and growth in a woody tropical bamboo from the brazilian atlantic forest. *New Zeal. J. Bot.* 51 275–285. 10.1080/0028825X.2013.829502

[B20] Holopainen J. K. (2002). Aphid response to elevated ozone and CO_2_. *Entomol. Exp. Appl.* 104 137–142. 10.1046/j.1570-7458.2002.01000.x

[B21] Howe G. A. Jander G. (2008). Plant immunity to insect herbivores. *Annu. Rev. Plant Biol.* 59 41–66. 10.1146/annurev.arplant.59.032607.092825 18031220

[B22] Huang M. Y. Huang W. D. Chou H. M. Chen C. C. Chang Y. T. Yang C. M. (2014). Herbivorous insects alter the chlorophyll metabolism of galls on host plants. *J. Asia Pacific Entomol.* 17 431–434. 10.1016/j.aspen.2014.04.004

[B23] Inbar M. Wink M. Wool D. (2004). The evolution of host plant manipulation by insects: molecular and ecological evidence from gall-forming aphids on pistacia. *Mol. Phylogenet. Evol.* 32 504–511. 10.1016/j.ympev.2004.01.006 15223033

[B24] Jablonski L. M. Wang X. Curtis P. S. (2002). Plant reproduction under elevated Co_2_ conditions: a meta-analysis of reports on 79 crop and wild species. *New Phytol.* 156 9–26. 10.1046/j.1469-8137.2002.00494.x

[B25] Kar P. K. Jena K. B. Srivastava A. K. Giri S. Sinha M. K. (2013). Gall-induced stress in the leaves of terminalia arjuna, food plant of tropical tasar silkworm, antheraea mylitta. *Emirtes J. Food Agricult.* 25 205–210. 10.9755/ejfa.v25i3.10970

[B26] Kmieć K. Rubinowska K. W. Michałek W. Sytykiewicz H. (2018). The effect of galling aphids feeding on photosynthesis photochemistry of elm trees (ulmus sp.). *Photosynthetica* 56 989–997. 10.1007/s11099-018-0813-9

[B27] Koyama Y. Yao I. Akimoto S. I. (2004). Aphid galls accumulate high concentrations of amino acids: a support for the nutrition hypothesis for gall formation. *Entomol. Exp. Appl.* 113 35–44. 10.1111/j.0013-8703.2004.00207.x

[B28] Kurzfeld-Zexer L. Wool D. Inbar M. (2010). Modification of tree architecture by a gall-forming aphid. *Trees* 24 13–18. 10.1007/s00468-009-0374-4

[B29] Kutsukake M. Meng X. Y. Katayama N. Nikoh N. Shibao H. Fukatsu T. (2012). An insect-induced novel plant phenotype for sustaining social life in a closed system. *Nat. Commun.* 3:1187 10.1038/ncomms2187www.nature.com/PMC3514493 23149732

[B30] Larson K. C. Whitham T. G. (1991). Manipulation of food resources by a gall-forming aphid: the physiology of sink-source interactions. *Oecologia* 88 15–21. 10.1007/bf00328398 28312726

[B31] Larson K. C. Whitham T. G. (1997). Competition between gall aphids and natural plant sinks: plant architecture affects resistance to galling. *Oecologia* 109 575–582. 10.1007/s004420050119 28307342

[B32] Liu P. Yang Z. X. Chen X. M. Foottit R. G. (2014). The effect of the gall-forming aphid *Schlechtendalia chinensis* (hemiptera: aphididae) on leaf wing ontogenesis in *Rhus chinensis* (sapindales: anacardiaceae). *Ann. Entomol. Soc. Am.* 107 242–250. 10.1603/AN13118

[B33] Lu Q. Chen H. Wang C. Yang Z. X. Lü P. Chen M. S. (2019). Macro- and Microscopic Analyses of Anatomical Structures of Chinese Gallnuts and Their Functional Adaptation. *Sci. Rep.* 9:5193 10.1038/s41598-019-41651 PMC6435719 30914739

[B34] Martin R. E. Asner G. P. Sack L. (2007). Genetic variation in leaf pigment, optical and photosynthetic function among diverse phenotypes of metrosideros polymorpha grown in a common garden. *Oecologia* 151 387–400. 10.1007/s00442-006-0604-z 17124568

[B35] Masle J. (2000). The effects of elevated CO_2_ concentrations on cell division rates, growth patterns, and blade anatomy in young wheat plants are modulated by factors related to leaf position, vernalization, and genotype. *Plant Physiol.* 122 1399–1416. 10.1104/pp.122.4.1399 10759536 PMC58975

[B36] Morkunas I. Mai V. C. Gabry B. (2011). Phytohormonal signaling in plant responses to aphid feeding. *Acta Physiol. Plant.* 33 2057–2073. 10.1007/s11738-011-0751-7

[B37] Newman J. A. Gibson D. J. Parsons A. J. Thornley J. H. M. (2003). How predictable are aphid population responses to elevated CO_2_? *J. Anim. Ecol.* 72 556–566. 10.1046/j.1365-2656.2003.00725.x 30893971

[B38] Patankar R. Thomas S. C. Smith S. M. (2011). A gall-inducing arthropod drives declines in canopy tree photosynthesis. *Oecologia* 167 701–709. 10.2307/41499982 21618011

[B39] Peltonen P. A. Julkunen-Tiitto R. Vapaavuori E. (2006). Effects of elevated carbon dioxide and ozone on aphid oviposition preference and birch bud exudate phenolics. *Glob. Change Biol.* 12 1670–1679. 10.1111/j.1365-2486.2006.01226.x

[B40] Pike N. Richard D. Foster W. Mahadevan L. (2002). How aphids lose their marbles. *Proc. R. Soc. Lond. B* 269 1211–1215. 10.1098/rspb.2002.1999 12065036 PMC1691028

[B41] Price P. W. Fernandes G. W. Waring G. L. (1987). Adaptive nature of insect galls. *Environ. Entomol.* 16 15–24. 10.1093/ee/16.1.15

[B42] Quintero C. Garibaldi L. A. Grez A. Polidori C. Nieves-Aldrey J. L. (2014). “Galls of the temperate forest of southern South America: argentina and chile,” in *Neotropical Insect Galls*, eds Fernandes G. W. Santos J. C. (Berlin: Springer Netherlands), 429–463. 10.1007/978-94-017-8783-3_21

[B43] Raman A. (2011). “Insect–plant interactions: the gall factor,” in *All Flesh Is Grass*, eds Dubinsky Z. Seckbach J. (Berlin: Springer Netherlands), 119–146. 10.1007/978-90-481-9316-5_5

[B44] Raskin I. Turner I. M. Melander W. R. (1989). Regulation of heat production in the inflorescences of an Arum lily by endogenous salicylic acid. *Proc. Natl. Acad. Sci. U.S.A.* 86 2214–2218. 10.1073/pnas.86.7.2214 16594020 PMC286882

[B45] Shao S. X. Yang Z. X. Chen X. M. (2013). Gall development and clone dynamics of the galling aphid *Schlechtendalia chinensis* (hemiptera: pemphigidae). *J. Econ. Entomol.* 106 1628–1637. 10.1603/EC13114 24020275

[B46] Smith S. D. Al E. (2000). Elevated CO_2_ increases productivity and invasive species success in an arid ecosystem. *Nature* 408 79–82. 10.1038/35040544 11081510

[B47] Thompson G. A. Goggin F. L. (2006). Transcriptomics and functional genomics of plant defence induction by phloem-feeding insects. *J. Exp. Bot.* 57 755–766. 10.1093/jxb/erj135 16495409

[B48] Tissue D. T. Thomas R. B. Strain B. R. (2010). Atmospheric CO_2_ enrichment increases growth and photosynthesis of pinus taeda: a 4 year experiment in the field. *Plant Cell Environ.* 20 1123–1134. 10.1046/j.1365-3040.1997.d01-140.x

[B49] Tooker J. F. Rohr J. R. Abrahamson W. G. Moraes C. M. D. (2008). Gall insects can avoid and alter indirect plant defenses. *New Phytol.* 178 657–671. 10.1111/j.1469-8137.2008.02392.x 18331430

[B50] Veteli T. O. Kuokkanen K. Julkunen-Tiitto R. Roininen H. Tahvanainen J. (2002). Effects of elevated CO_2_ and temperature on plant growth and herbivore defensive chemistry. *Glob. Change Biol.* 8 1240–1252. 10.1046/j.1365-2486.2002.00553.x

[B51] Vólkl W. Woodring J. Fischer M. Lorenz M. W. Hoffmann K. H. (1999). Ant-aphid mutualisms: the impact of honeydew production, and honeydew sugar composition on ant preferences. *Oecologia* 118 483–491. 10.1007/s004420050751 28307416

[B52] Wang H. Y. Liu J. Cui K. Chen H. Yang Z. X. Wu H. X. (2016). Gibberellic acid is selectively downregulated in response to aphid-induced gall formation. *Acta Physiol. Plant.* 38:214 10.1007/s11738-016-2224-5

[B53] Wang S. C. Xu L. L. Li G. J. Chen P. Y. Xia K. Zhou X. (2002). An ELISA for the determination of salicylic acid in plants using a monoclonal antibody. *Plant Sci.* 162 529–535. 10.1016/s0168-9452(01)00606-9

[B54] Will R. E. Ceulemans R. (2006). Effects of elevated CO_2_ concentration on photosynthesis, respiration and carbohydrate status of coppice populus hybrids. *Physiol. Plant.* 100 933–939. 10.1111/j.1399-3054.1997.tb00020.x

[B55] Wool D. (2004). Galling aphids: specialization, biological complexity, and variation. *Annu. Rev. Entomol.* 49 175–192. 10.1146/annurev.ento.49.061802.123236 14651461

